# Valvular Heart Disease in Indian Subcontinent: Social Issues

**DOI:** 10.4103/0970-0218.45374

**Published:** 2009-01

**Authors:** Ramachandran Meenakshisundaram, Ponniah Thirumalaikolundusubramanian

**Affiliations:** Institute of Internal Medicine, Madras Medical College, Chennai – 600003, India

## Introduction

Rheumatic heart disease (RHD) is more prevalent in underdeveloped and developing countries than in developed countries(1) and among the population with multiple social issues such as poverty, low socio-economic status (SES), overcrowded dwellings, under-nutrition, poor sanitation,(1) cultural constraints,(2) and suboptimal medical care.(3) Published reports on RHD from India and elsewhere focused more on prevalence,(1,3,4) socio-demographic factors,(1–4) pattern of illness,(5) presenting features, clinical course, management aspects,(1) and complications.(6) In this brief article, some of the social issues related to mitral stenosis along with clinical aspects are discussed and suggestions are provided.

## Materials and Methods

One hundred patients with isolated rheumatic mitral stenosis who underwent mitral commisurotomy and are attending the hospital either for follow-up or complications and another 100 new cases of the same confirmed by echocardiogram were included in this study. Individuals who were pregnant or had anemia or other coexisting illnesses were excluded. Patients were interviewed first by the author in the local vernacular with a structured questionnaire to elicit socio-demographic details, clinical aspects, acceptance of treatment, and other social issues faced by them. The study was carried out from October 2006 to May 2007 after institutional ethical clearance and informed consent from patients.

Socioeconomic status was assessed as per the guidelines provided by Aggarwal, *et al*.(7) As per the American heritage dictionary, compliance is defined as the adherence to the drug regimen in taking medication correctly and on time. It encompasses the patient's active participation in his or her own health care; seeking medical advice, keeping appointments, following recommendations concerning lifestyles as well as following medical regimens. Data were entered in a Microsoft Excel^®^ Worksheet and analyzed by simple descriptive statistics.

## Results

There were 64 males and 36 females in the operated group, and among the newly diagnosed cases, 42 were males and 58 were females. The mean ages of the former and later groups were 23 and 34 years respectively. The rural to urban ratio was 4:1. All the patients belonged to a low SES and were living in crowded areas with poor hygiene. Various complications noticed among operated and new cases were congestive heart failure (24;54%), acute pulmonary edema (13;31%), arrhythmias (29;42%), embolic episodes (14;21%), and infective endocarditis (0;3%). Within a period of 3 to 5 years, 29% of the operated cases developed symptoms and signs of restenosis. Difficulties encountered by operated and new cases were financial constraints for transport (70;56%), lack of family support (38;31%) and fear of drugs are provided expenses (59;38%). Nearly one-third of the patients preferred complimentary and alternative medicine (24;31%). During follow-up, non-compliance was noticed among 32% of the operated cases and 15% of the new cases. When surgery was recommended to all the new cases, 37% accepted surgery (23 females and 14 males), 30% refused surgery (10 females and 20 males), and 33% could not make a decision (25 females and 8 males).

## Discussion

In India, RHD is prevalent among children and young adults. Despite existing free school health services and regular ante-natal check-up programs at all levels of government hospital/health centers, many cases were not diagnosed before overt manifestations or complications. Since these patients continue to suffer from the illness, their productivity is lost, which imposes an economical burden on their family and country.

Patients with RHD were motivated to get secondary prophylaxis from the nearest health center and asked to enter the details in the follow up card, which was verified during their review. At the same time, getting drugs (free of cost) in our hospital on weekly basis caused financial burden and personal inconvenience. Due to family support and encouragement from their husbands, the acceptance rate for surgery was significantly higher among females. Of the males, 50% refused surgery due to fear, illiteracy, or lack of social support. Even if the patients are willing to undergo surgery, they have to wait for 2 to 3 years. Restenosis is not uncommon after mitral commisuratomy, a preferred mode of treatment in this hospital and elsewhere; but onset varies with surgical skills, place of surgery, healing process, genetic factors for fibrosis, pattern of living, and compliance.

A long waiting time in government hospitals for surgery among patients with restenosis and new cases (29 and 100) is due to the lack of required surgeons and limited facilities. Hence, the State Government of Tamil Nadu has a collaboration with leading, accredited private hospitals for cardiac surgery, which reduces a long waiting time and is working well.(8) This is a significant venture in Public-Private Partnership and is a model for others. Funding has been made available from the Tamil Nadu Chief Minister's welfare fund and disbursed through the District Collector to the respective hospitals for surgery.

### IEC messages for patients with RHD

To overcome social issues, awareness about RHD (source, transmission, clinical course, and complications) should be created among individuals and family members through Information, Education, and Communication. Patients should also be educated about personal and environmental hygiene, secondary prophylaxis, lifestyle modification, and the importance of regular follow-up. All pregnant women should be examined for RHD during their ante-natal visits and entries may be made in records and reviewed. Communication activities can be mediated by counselors, social workers, health visitors, village health nurses, and medical officers of health centers individually or in a group through role play, story telling, audiovisual aids, posters, handbills, flash cards, folders and flip charts,(9) and through information technology,(10) which may be extended through school health services.

It is suggested that improvement in the standard of living and health care facilities, provision of transport concessions, creation of awareness through media regarding RHD and strengthening of school health services will assist to minimize the occurrence, which could be achieved through National Health Policy, National Rural Health Mission, and Public-Private Partnerships.

The strength of this study is that all the cases were confirmed by echocardiogram. The limitations of this study were a lack of regular echocardiograms during follow-up for operated cases and selection limited to mitral stenosis.

## References

[1] Periwal KL, Gupta BK, Panwar RB, Khatri PC, Raja S, Gupta R (2006). Prevalence of rheumatic heart disease in school children in Bikaner: An echocardiographic study. J Assoc Physicians India.

[2] Mincham CM, Toussaint S, Mak BD, Plant AJ (2003). Patient views on the management of rheumatic fever and rheumatic heart disease in the Kimberley: A qualitative study. Aust J Rural Health.

[3] Longo-Mbenza B, Bayekula M, Ngiyulu R, Kintoki VE, Bikangi NF, Seghers KV (1998). Survey of rheumatic heart disease in school children of Kinshasa town. Int J Cardiol.

[4] Rizvi SF, Khan MA, Kundi A, Marsh DR, Samad A, Pasha O (2004). Status of rheumatic heart disease in rural Pakistan. Heart.

[5] Misra M, Mittal M, Singh RK, Verma AM, Rai R, Chandra G (2007). Prevalence of rheumatic heart disease in school-going children of eastern Uttar Pradesh. Indian Heart J.

[6] Akdemir I, Dagdelen S, Yuce M, Davutoglu V, Akcay M, Akdemir N (2002). Silent brain infarction in patients with rheumatic mitral stenosis. Jpn Heart J.

[7] Aggarwal OP, Bashin SK, Sharma AK, Chaabre P, Aggarwal K, Rajouse OP (2005). A new instrument (scale) for measuring socioeconomic status of a family: Preliminary study. Indian J Com Medicine.

[8] (2008). Beneficiaries of free heart surgery scheme thank Karunanidhi. The Hindu, Madurai.

[9] Jai Vigyan Mission mode Project on control of rheumatic fever and rheumatic heart disease Prevention, diagnosis and management of RF/RHD. Guidelines for doctors.

[10] Padmavati S (2002). Rheumatic heart disease in the SAARC countries: Past, present and future directions. J Cardiol.

